# Effect of COVID‐19 infection on the gastrointestinal tract considering preventive methods during endoscopic procedures

**DOI:** 10.1002/deo2.290

**Published:** 2023-08-27

**Authors:** Sakiko Naito, Itaru Nakamura, Tomohiro Umezu, Akihiro Hata, Akira Madarame, Kumiko Uchida, Yohei Koyama, Takashi Morise, Hayato Yamaguchi, Shin Kono, Mitsushige Sugimoto, Takashi Kawai, Yuichiro Harada, Masahiko Kuroda, Masakatsu Fukuzawa, Takao Itoi

**Affiliations:** ^1^ Department of Gastroenterology and Hepatology Tokyo Medical University Tokyo Japan; ^2^ Department of Infection Control and Prevention Tokyo Medical University Tokyo Japan; ^3^ Department of Molecular Pathology Tokyo Medical University Tokyo Japan; ^4^ Department of Gastroenterological Endoscopy Tokyo Medical University Tokyo Japan

**Keywords:** COVID‐19, digestive fluid infection, endoscopy, fecal PCR, gastrointestinal tract

## Abstract

**Objectives:**

This study aimed to prevent the infection risk of environmental contamination by feces during endoscopic procedures. We evaluated the effect of coronavirus disease 2019 (COVID‐19) on the gastrointestinal tract using fecal polymerase chain reaction (PCR) and examined risk factors affecting endoscopic procedures, to develop infection prevention strategies.

**Methods:**

This single‐center prospective observational study enrolled 32 patients diagnosed with COVID‐19 at Tokyo Medical University Hospital between January and December 2022. We performed reverse transcriptase‐PCR to detect severe acute respiratory syndrome coronavirus 2 in human stool specimens and evaluated the COVID‐19 positivity rate in stool, the effect of vaccination on infection detection, and differences in positivity rates considering different patient backgrounds.

**Results:**

Among the 32 nasal PCR‐positive patients who underwent fecal PCR testing, the fecal PCR positivity rate was 21.8%. Compared to the negative cases, 71.4% vs. 32% were older than 65 years (*p* < 0.016), 71.4% vs. 0.8% (*p* < 0.001) had malignant tumors, the rate during BA.5 variant outbreaks was significantly higher (100% vs. 60% [*p* = 0.044]), and the rate of diarrheal symptoms was also higher (42.9% vs. 24%). The median collection period for fecal PCR‐positive cases was 2 days after sampling.

**Conclusions:**

The severe acute respiratory syndrome coronavirus 2 affects not only the upper respiratory tract but also the gastrointestinal tract. These findings may indicate the risk of digestive fluid infection in older patients with gastrointestinal symptoms and immunocompromised patients with malignant tumor comorbidities, especially during the early stages of viral infection. Therefore, it is advisable to establish a system to prevent infection by using personal protective equipment, including eye guards, in future endoscopic procedures.

## INTRODUCTION

Severe acute respiratory syndrome coronavirus 2 (SARS‐CoV‐2), which originated in Wuhan, China, in January 2019, caused a pandemic owing to its mutation and high infection rate.^1^ According to reports from the World Health Organization and Centers for Disease Control and Prevention, SARS‐CoV‐2, the causative virus of coronavirus disease 2019 (COVID‐19), can be spread by aerosol transmission through air, contaminated fingers, and indirect contact transmission through the environment.^2^, ^3^ It has been reported that aerosol droplets can spread over wide areas during endoscopic procedures, including the surgeon's mask and gloves; hence, personal protective equipment is recommended during endoscopic procedures. Miyake et al.^4^ reported that viruses were detected in digestive fluids such as gastric juice, feces, and even saliva of patients who are COVID‐19‐negative. In addition, environmental exposure to digestive solutions has been reported.^5^, ^6^, ^7^, ^8^ As SARS‐CoV‐2 enters target cells via angiotensin‐converting enzyme 2 receptors, which are frequently present in the lungs and gastrointestinal tract, it produces upper respiratory tract symptoms such as fever and cough and gastrointestinal symptoms such as nausea and diarrhea. This suggests that droplets from the airway and fecal secretions dispersed as aerosols are infectious.^9^, ^10^ The risk of environmental contamination via digestive juices is an important consideration in endoscopic procedures; however, the effects of digestive juices on gastrointestinal tract function remain unknown. Although there have been reports on measures to prevent environmental contamination,^11^ there are no frequent studies on infection risk measures, and it is important to perform infection triage by assessing risks, such as the timing of infection, differences in background factors, and symptoms, when preventing infection during endoscopic procedures involving digestive fluids. Therefore, this study aimed to evaluate the risk of infection in patients with COVID‐19 in association with gastrointestinal tract symptoms using fecal real‐time (rt)‐polymerase chain reaction (PCR) tests.

## METHODS

### Patients and study design

This was a single‐center prospective observational study involving 32 patients diagnosed with COVID‐19 at Tokyo Medical University Hospital between January and December 2022. This study was approved by the Institutional Review Board of the Tokyo Medical University Hospital (registration number: T2021‐0114). The study protocol conformed to the ethical guidelines of the Declaration of Helsinki 1964 as reflected in a priori approval by the human research committee of the institution. This study was registered with the University Hospital Medical Information Network Clinical Trials Registry. (www.umin.ac.jp/ctr/; identification no.: UMIN000051273) Because this was a prospective observational study and written informed consent was obtained from each patient, a document describing the opt‐out policy was uploaded to the Tokyo Medical University Hospital website.

### Fecal viral RNA extraction

**FIGURE 1:**
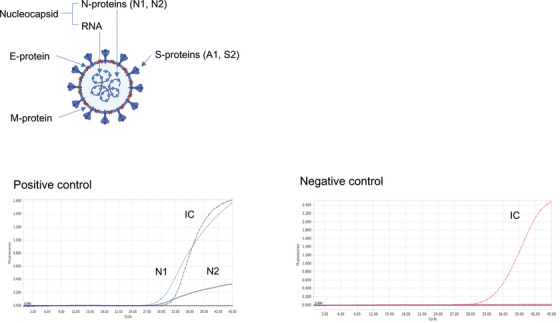
The sample solution was collected in a tube (Roche Light Cycler 96) and heated at 70°C for 15 min. Then, 7 μL of the sample solution was mixed with Reagent Kit 1 solution and heated at 90°C for 5 min. We detected two N (Nucleocapsid) genes (N1 and N2) of the SARS‐CoV‐2 virus and defined fecal polymerase chain reaction (PCR) positivity as positive for both N1 and N2.

We performed reverse‐transcriptase rt‐PCR for the detection of SARS‐CoV‐2 in human stool samples. Stool samples collected for fecal PCR were swabbed and stored at −80°C. After inactivation with MEBRIGHTTM Virus Inactivation Solution 2, the same Ampdirect 2019‐nCoV Detection Kit (Shimadzu Corporation, Kyoto, Japan) was used for the nasal samples. The sample solution was collected in a tube (Roche Light Cycler 96) and heated at 70°C for 15 min. Then, 7 μL of the sample solution was mixed with Reagent Kit 1 solution and heated at 90°C for 5 min. We detected two N(Nucleocapsid) genes (N1 and N2) of the SARS‐CoV‐2 virus and defined fecal PCR positivity as positive for both N1 and N2. (Figure 1). We used three colors of fluorescence for detection in the PCR in this test: the first color is the N1 region of the COVID‐19 nucleocapsid and the second color is the N2 region. The sample treatment reagent contains an internal control to confirm that the PCR is running well, which is detected and judged by the third color. Positive controls were evaluated using the positive control standard RNA for nCov. The cut‐off value was set at 38 cycles using the Light Cycler 96 for CoV RNA. Infection control measures were performed according to the biosafety level 2 guidelines.^12^ Patient background characteristics (age, sex, body mass index, rate of older patients, smoker, vaccination status, admission period, period of stool sample taking, medication, epidemic mutation, severity, symptoms, and comorbid disease) were assessed using medical records. We examined COVID‐19 positivity in stool, the effect of vaccines on infection detection, and differences in positivity rates due to varying patient backgrounds. A time‐series comparison was conducted for the number of new patients with positive and negative fecal test cases at Tokyo Medical University Hospital, which covers cases of COVID‐19 in the entire Tokyo metropolitan area and its prevalent strains. This information was extracted from the official website of the Tokyo Metropolitan Government (https://stopcovid19.metro.tokyo.lg.jp/).

### Classification of disease severity

Disease severity was determined according to the Ministry of Health, Labor, and Welfare's COVID‐19 Clinical Practice Guide (9th Edition; Figure S1).

Mild disease was defined as SpO_2_ ≥ 96%, no respiratory symptoms or only cough, no dyspnea, and no pneumonia. Moderate I disease was defined as 93% < SpO_2_ < 96%, with dyspnea and pneumonia present. Moderate II disease was defined as SpO_2_ ≤ 93%, with oxygen administration required for clinical symptoms. Severe disease was defined as that requiring intensive care unit admission.

### Statistical analysis

In this study, the COVID‐19 positivity rate by stool sample rt‐PCR was evaluated using the Chi‐square test or Fisher's exact test for differences (patient background, COVID‐19 vaccination history, and COVID‐19 detection in feces) between nasal and fecal rt‐PCR positive specimens.

All statistical analyses were performed using the SPSS software (version 27.0; IBM Japan), and a *p*‐value <0.05 was considered statistically significant.

## RESULTS

### Patient characteristics

The median age was 50 (interquartile range [IQR]: 33–72) years, the proportion of male patients was 68.8%, the median temperature was 38 (IQR: 37–39)°C, vaccination history was reported in 90.6%, and median fecal sample collection period was 5 (IQR: 2–9) days. In total, 40.6% of the patients had a treatment history: 3.1% antibiotics, 9.3% steroids, and 27.9% antivirals. Mutant strains of Omicron strain Ba.5 and fecal PCR positivity were 68.8% and 21.9%, respectively. The severity of the disease was mild in 87.5% of cases. Fever of ≥38°C and upper respiratory tract symptoms were present at a high rate (62.5% and 62.5%), and diarrhea symptoms were present in 27.9% of the cases overall.

Asymptomatic cases were also observed in 12.5% of the patients. The following comorbidities were reported: respiratory disease (6.3%), cardiovascular disease (9.4%), diabetes mellitus (9.4%), chronic renal dysfunction (3.1%), gastrointestinal disease (28.1%), liver disease (6.2%), inflammatory bowel disease (6.2%), hematological disease (9.4%), gastrointestinal cancer 15.6%), and malignant tumors (21.8%; Table 1).

**TABLE 1:** Characteristics of patients with coronavirus disease 2019.

	*n* = 32
Age; median (IQR), years	50 (33–72)
Sex (M/F)	22/10
BMI, median (IQR), years	21.8 (19.6–23.2)
Older patients ≥65, *n* (%)	12 (37.5)
Smoker (current), *n* (%)	2 (6.3)
Vaccination status, *n* (%)	29 (90.6)
Admission period, median (IQR)	6 (4–8)
Period of stool taking, median (IQR)	5 (2–9)
Medication, *n* (%)	
PPI, *n* (%)	6 (25)
Treatment, *n* (%)	13 (40.6)
Antibiotics, *n* (%)	1 (3.1)
Steroid, *n* (%)	3 (9.3)
Molnupiravir, *n* (%)	9 (27.9)
Epidemic mutation of Ba.5, *n* (%)	22 (68.8)
rt‐PCR positive, *n* (%)	7 (21.9)
rt‐PCR positive on only one side, *n* (%)	5 (15.6)
Severity, mild/severe, *n* (%)	3 (9.3)
Symptoms, *n* (%)	
Fever, median (IQR)	38 (37–39)
Pharyngalgia, *n* (%)	20 (60.2)
Dyspnea, *n* (%)	6 (18.6)
Coughing, *n* (%)	19 (59.4)
Diarrhea, *n* (%)	9 (27.9)
Asymptomatic, *n* (%)	4 (12.5)
Disease	
Diabetes mellitus, *n* (%)	3 (9.4)
Chronic respiratory disease, *n* (%)	2 (6.3)
Asthma, *n* (%)	1 (3.1)
Heart disease, *n* (%)	3 (9.4)
Hypertension, *n* (%)	3 (9.4)
Dyslipidemia, *n* (%)	2 (6.3)
Chronic liver disease, *n* (%)	3 (9.4)
Malignant tumor, *n* (%)	7 (21.8)
Immunosuppressed, *n* (%)	5 (15.6)
HIV, *n* (%)	1 (3.1)
IBD, *n* (%)	2 (6.2)
Hematological disease, *n* (%)	2 (6.2)

Abbreviations: HIV, human immunodeficiency virus; IBD, inflammatory bowel disease; IQR, interquartile range; PPI, proton pump inhibitor.

### Variation in background factors in fecal PCR‐positive patients

**FIGURE 2:**
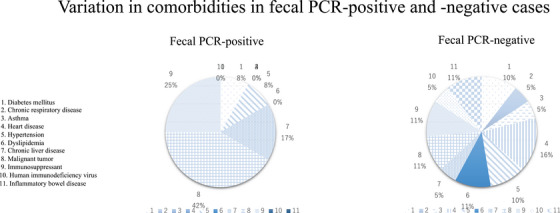
Variation in comorbidities in fecal polymerase chain reaction (PCR)‐positive and ‐negative cases. There was a high rate of malignant disease in fecal PCR‐positive cases.

A significantly higher rate (*p* = 0.016) of fecal PCR‐positive cases was found in older patients aged (median) 71 years (IQR 66–72) and diarrhea was present in 47.9% of gastrointestinal symptoms. Three cases of advanced esophageal cancer, two cases of liver cancer, and one case of chronic pancreatic disease were noted for gastrointestinal diseases. A significantly higher proportion of the positive patients had cancer (71.4%, *p* < 0.001; Figure 2), and the prevalent mutant virus was the Omicron strain BA.5 in all cases (*p* = 0.044). There was no association between proton pump inhibitor administration and fecal PCR, and no difference in vaccination history between the negative and positive PCR groups (71.4% vs. 84%). Fecal samples were collected for 2 days after the onset of COVID‐19 in the rt‐PCR‐positive cases and 6 days after infection in the rt‐PCR‐negative cases. Patients with rt‐PCR‐positive stool samples were hospitalized for slightly longer periods. Among the rt‐PCR‐negative cases, some patients were not hospitalized but were managed at home. rt‐PCR‐positive cases were treated with antivirals at a high rate.

Cough and diarrhea symptoms were observed in a high percentage (57.1% vs. 25% and 42.9% vs. 24%, respectively) of fecal rt‐PCR‐positive cases compared to rt‐PCR‐negative cases, while asymptomatic cases were observed in a high percentage (0% vs. 16%) of fecal rt‐PCR‐negative cases compared to rt‐PCR‐positive cases. (Table 2).

**TABLE 2:** Clinical characteristics of fecal real‐time polymerase chain reaction‐positive and negative patients.

	Fecal rt‐PCR positive (*n* = 7)	Fecal rt‐PCR negative (*n* = 25)	*p‐*Value
Age, median (IQR), years	71 (66–72)	44 (32–72)	0.017
Sex (M/F)	3/4	4/21	0.076
BMI, kg/m^2^	21.6 (19.8–24.2)	21.8 (19.8–23.1)	0.651
Older people ≥65, *n* (%)	5 (71.4)	8 (32)	0.016
Smoker (current), *n* (%)	0	2 (8)	0.440
Vaccination status, *n* (%)	5 (71.4)	21 (84)	0.557
Admission period, median (IQR)	13 (6–21)	5 (4–7)	0.651
Period of stool sample taking, median (IQR)	2 (2–5)	6 (3–13)	0.169
Medication, *n* (%)			
PPI, *n* (%)	3 (42.9)	3 (12)	0.169
Treatment, *n* (%)	3 (42.9)	10 (40)	0.765
Antibiotics, *n* (%)	0	1 (4)	0.590
Steroid, *n* (%)	0	3 (12)	0.336
Molnupiravir, *n* (%)	3 (42.9)	6 (24)	0.327
Epidemic mutation of Ba.5, *n* (%)	7 (100)	15 (60)	0.044
Severity, mild/moderate/severe, *n*	6/1/0	22/3/0	0.807
Symptoms			
Fever, median (IQR)	38 (38–38.5)	38 (37–38)	0.247
Pharyngalgia, *n* (%)	4 (57.1)	15 (60)	0.683
Dyspnea, *n* (%)	1 (14.3)	5 (20)	0.837
Coughing, *n* (%)	4 (57.1)	5 (20)	0.145
Diarrhea, *n* (%)	3 (42.9)	6 (24)	0.327
Asymptomatic, *n* (%)	0	4 (16)	0.627
Disease			
Diabetes mellitus, *n* (%)	1 (14.3)	2 (8)	0.536
Chronic respiratory disease, *n* (%)	0	1 (4)	0.590
Asthma, *n* (%)	0	1 (4)	0.590
Heart disease, *n* (%)	0	3 (12)	0.336
Hypertension, *n* (%)	1 (14.3)	2 (8)	0.536
Dyslipidemia, *n* (%)	0	2 (8)	0.440
Chronic liver disease, *n* (%)	2 (28.6)	1 (4)	0.113
Malignant tumor, *n* (%)	5 (71.4)	2 (8)	<0.001
Immunosuppressed, *n* (%)	3 (42.9)	2 (8)	0.057
HIV, *n* (%)	0	1 (4)	0.590
IBD, *n* (%)	0	2 (8)	0.912

Abbreviations: BMI, body mass index; HIV, human immunodeficiency virus; IBD, inflammatory bowel disease; IQR, interquartile range; PPI, proton pump inhibitor; rt‐PCR, real‐time polymerase chain reaction.

### Summary of rt‐PCR in stool specimens

We performed reverse‐transcriptase rt‐PCR for the detection of SARS‐CoV‐2 in human stool samples. We detected two N genes (N1 and N2) of the SARS‐CoV‐2 virus and defined fecal PCR positivity as positive for both N1 and N2. Of the 32 cases, seven (21.8%) were positive. There were five cases in which only one side was positive, two of which were inflammatory bowel disease, one of which was chronic pancreatitis, one of which was a hematological disease, and one case had no comorbidities (Table 3).

**TABLE 3:** Summary of real‐time polymerase chain reaction in stool specimens.

Patient	1	2	3	4	5	6	7	8	9					
COVID‐19 (N1)	‐	‐	‐	‐	‐	‐	‐	‐	‐					
COVID19 (N2)	‐	‐	‐	‐	‐	‐	‐	‐	‐					
Control	31.72	31.34	31.72	31.95	32.68	31.05	32.14	31.76	32.43					
Patient	10	11	12	13	14	15	16	17	18	19	20	21	22	23
COVID‐19 (N1)	‐	‐	‐	‐	36.84	33.52	‐	30.8	40.61	33.13	‐	‐	‐	33.32
COVID‐19 (N2)	‐	‐	‐	‐	‐	‐	‐	32.5	‐	38.71	‐	‐	‐	35.65
Control	31.72	30.62	31.34	30.52	31.85	31.66	32.08	31.17	32.15	31.51	31.73	31.71	31.71	31.49
Patient	24	25	26	27	28									
COVID‐19 (N1)	‐	35.94	30.13	‐	33.61									
COVID‐19 (N2)	‐	‐	31.11	‐	34.23									
Control	31.8	31.15	30.77	31.8	31.7									
Patient	29	30												
COVID‐19 (N1)	32.94	39.18												
COVID‐19 (N2)	33.84	‐												
Control	31.83	32.03												
Patient	31	32												
COVID‐19 (N1)	‐	31.6												
COVID‐19 (N2)	‐	33.01												
Control	31.76	32.75												

Abbreviation: COVID‐19, coronavirus disease 2019.

### Association of fecal rt‐PCR cases with COVID‐19 prevalence trends

**FIGURE 3:**
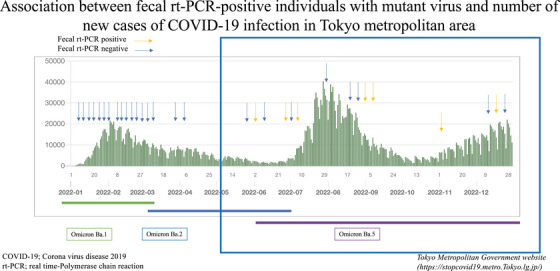
The relationship between the number of new infections and the time of sampling, mutant strain, and positive cases in the Tokyo metropolitan area. The fecal real‐time polymerase chain reaction (rt‐PCR)‐positive cases occurred during the time of the Omicron strain outbreak.

The number of new infections in the Tokyo metropolitan area and the relationship between sampling, mutant strain, and number of positive cases are shown in Figure 3.

Fecal rt‐PCR‐positive cases were observed coinciding with the time of the Omicron strain Ba.5 epidemic.

## DISCUSSION

### Effects of SARS‐CoV‐2 on gastrointestinal tract dysfunction

SARS‐CoV‐2 invades target cells via the angiotensin‐converting enzyme 2 receptor, especially in immunocompromised patients, and it is thought to cause a cytokine storm, which increases intestinal permeability and causes persistent infection, resulting in gastrointestinal symptoms such as diarrhea.^13^, ^14^ The mechanism of this effect is that the virus particles infect cells and, similar to SARS‐CoV, affect immune cells such as lymphocytes, especially T lymphocytes. This leads to a cytokine storm, which contributes to gastrointestinal tract inflammation, increasing intestinal permeability and contributing to persistent infection^15^ and, eventually, gastrointestinal symptoms such as diarrhea. It directly exacerbates symptoms and affects prognosis by the increased intestinal permeability due to cytotoxicity, abnormal intestinal bacteria, and abnormal immune response. This specific immune response has been shown to cause a propensity for severe disease in patients with diarrheal symptoms because of its effect on viral evacuation.^16^, ^17^, ^18^ In the present study, fecal PCR results were significantly positive among older patients (*p* = 0.016). Aging is considered a risk factor for severe disease owing to decreased immune function; hence, older individuals are more susceptible to severe disease.^9^ In addition, fecal PCR positivity was frequently observed in patients presenting with gastrointestinal symptoms, especially in 71.4% of patients with digestive carcinoma (*p* < 0.001; Table 1). In asymptomatic cases, the fecal PCR test is negative (Table 1), and cytokine storms are not considered adverse and may have little influence on the intestinal tract.

### Effects of vaccination on the gastrointestinal tract

The results of the present study are consistent with that of a previous study^19^ wherein diarrheal symptoms were more frequently observed in immunocompromised patients. Of the fecal PCR‐positive patients, the median age of the patients was 71 years, which was older than that of the negative cases, 66.7% had gastrointestinal carcinoma and were treated with chemotherapy (Table 2). Because of the significantly lower diversity in immunocompromised patients than in healthy controls, there is a decrease in the proportion of beneficial commensal bacteria and an increase in opportunistic pathogens.^19^, ^20^

In this study, patients with PCR‐positive fecal samples were vaccinated; however, their compromised immunity may have caused a difference in antibody retention, resulting in a cytokine storm and persistent infection of the intestinal tract. However, we cannot rule out the possibility that decreased cytokine storm due to vaccination‐induced acquired immunity^21^ may have contributed to the negative results, especially in the five patients who tested positive on only one side.

Gastrointestinal symptoms and frequent diarrhea in immunocompromised COVID‐19‐positive patients may have severe presentations due to elevated intestinal permeability and should be adequately monitored. Moreover, even if a patient is vaccinated, there is a possibility of decreased efficacy of prophylaxis due to immune deficiencies. Furthermore, patients treated with chemotherapy, cisplatin, and other therapies are susceptible to not only COVID‐19 but also other severe diseases.^22^ When immunocompromised patients present with gastrointestinal symptoms, which serve as predictors of prognosis, additional investigation of background factors for COVID‐19 should be considered. This holds true even if there is a history of immunization.

### Association with mutant viral strains

According to the World Health Organization, B.1.1.7 (alpha strain), B.1.351 (beta strain), B.1.6172.2 (delta strain), and B.1.1.529 (Omicron strain) virus strains cause different mutations and allow different transmission and symptoms. The present study was conducted on cases diagnosed from January to December 2022. According to the National Institute of Infectious Diseases, the major mutant viral strains in Japan during this period were Omicron BA.1, 2, and BA.5. Because the fecal PCR‐positive patients were also positive for the Omicron BA.5 mutant strain during the pandemic, the mutant virus strain may affect the gastrointestinal tract. New outbreaks may occur in the future owing to new mutant strains and the subsequent decreased efficacy of the previously made vaccines.^23^ Immunocompromised patients are characterized by severe symptoms and administration of chemotherapy, and the conversion process is variable.^22^ Long‐term infection in immunocompromised patients has led to the occurrence of mutant strains. Therefore, it is important to focus on infection control measures to prevent disease spread. Omicron strains have been reported to have a short period of disease incubation (median 3.42 days; 95% confidence interval 2.88–3.96).^24^ Since the period of the Omicron strain outbreak reported in this study was also the period of the spread of Omicron, it is possible that the viral effect on the intestinal tract was observed for a short period; hence, there is a need to observe the characteristics of new viral mutant strains in the future to establish infection prevention strategies.

### Prevention strategy against infection in endoscopy

COVID‐19 has been implicated in the aerosol transmission of COVID‐19 through air.^5^ There are appropriate precautions that can be taken by using personal protective equipment to prevent the spread of the virus,^8^ considering the aerosol generation due to the frequent viral load in the lower respiratory tract.^25^ Also, during endoscopic examinations, there was widespread droplet contamination not only by the masks and gloves of the surgeons but also from their medical caps,^11^ indicating that aerosol infection control strategies are required. However, there have been no reports of environmental contamination during colonoscopic procedures, viruses have been detected in feces.^26^ Fecal infection is possible because SARS‐CoV‐2 stays in the intestinal tract for 11–12 days.^27^ In our study, the median time of negative PCR test result was 6 days after infection, suggesting that the virus affects the intestinal tract for a shorter period than previously reported or that it may not affect the intestinal tract. Moreover, many cases are not accompanied by gastrointestinal symptoms, the virus may not penetrate the intestinal tract. Considering that the virus is found in the feces of COVID‐19‐positive patients, the effect of infectious aerosols from feces as well as aerosols associated with coughing due to upper respiratory tract infection should be noted during endoscopic examination in patients with COVID‐19. Therefore, fecal PCR positivity occurring (median) 2 days after infection and fecal PCR negativity occurring 6 days after possible infection may serve as an indicator for preventive measures. According to a previous study,^27^ infection is determined by the duration of viral exposure and the amount of virus, and there is a longer period of viral shedding from the feces than from the upper respiratory tract.^13^, ^27^ It is necessary to take preventive measures against the possibility of fecal positivity in patients with gastrointestinal symptoms for a certain period after infection (2–3 days), as the virus is strongly contagious in patients with gastrointestinal symptoms.^28^ Therefore, it is important to improve the environment for appropriate endoscopic examination and continue screening to ensure early detection and treatment of disease.

This study has some limitations. First, this was a single‐institution, prospective study. Second, this study did not confirm negative results in fecal PCR‐positive patients, which precludes an accurate definition of negative results. Third, the fecal collection time for PCR varied for individual samples in each case, which may have resulted in differences in the effect of the virus on the gastrointestinal tract. Some PCR‐negative individuals might have tested positive if the sampling had been performed earlier. Fourth, further investigation of viral inactivation in the intestinal tracts of asymptomatic or symptomatic patients who are COVID‐19‐positive without gastrointestinal symptoms is required. Different viral variants may have varying effects on the gastrointestinal tract owing to the limited time of fecal sample collection.

In conclusion, SARS‐CoV2 has been suggested to detect not only the upper respiratory tract but also the gastrointestinal tract. This may indicate the risk in older patients and immunocompromised patients with gastrointestinal symptoms, especially in the early stages of viral infection. The findings of the study imply the need for infection preventive measures by using personal protective equipment, including eye guards, in endoscopic procedures and the establishment of an endoscopic examination system.

## CONFLICT OF INTEREST STATEMENT

Takao Itoi is an Editor in Chief of *Digestive Endoscopy Open*. The other authors declare no conflict of interest.

## Supporting information

Figure S1 The severity of cases affected by COVID‐19 was evaluated according to the medical guide of the Ministry of Health, Labour, and Welfare.

## References

[1] Huang C , Wang Y , Li X *et al*. Clinical features of patients infected with 2019 novel coronavirus in Wuhan, China. Lancet 2020; 395: 497–506. 31986264 10.1016/S0140-6736(20)30183-5 PMC7159299

[2] Yu IT , Li Y , Wong TW *et al*. Evidence of airborne transmission of the severe acute respiratory syndrome virus. N Engl J Med 2004; 350: 1731–9. 15102999 10.1056/NEJMoa032867

[3] Rothan HA , Byrareddy SN . The epidemiology and pathogenesis of coronavirus disease (COVID‐19) outbreak. J Autoimmun 2020; 109: 102433. 32113704 10.1016/j.jaut.2020.102433 PMC7127067

[4] Miyake S , Ashikari K , Kato S *et al*. Severe acute respiratory syndrome coronavirus 2 prevalence in saliva and gastric and intestinal fluid in patients undergoing gastrointestinal endoscopy in coronavirus disease 2019 endemic areas: Prospective cross‐sectional study in Japan. Dig Endosc 2022; 34: 96–104. 33548095 10.1111/den.13945 PMC8014498

[5] Zhou J , Otter JA , Price JR *et al*. Investigating severe acute respiratory syndrome coronavirus 2 (SARS‐CoV‐2) surface and air contamination in an acute healthcare setting during the peak of the coronavirus disease 2019 (COVID‐19) pandemic in London. Clin Infect Dis 2021; 73: e1870–7. 32634826 10.1093/cid/ciaa905 PMC7454437

[6] Hoseinzadeh E , Javan S , Farzadkia M , Mohammadi F , Hossini H , Taghavi M . An updated mini‐review on environmental route of the SARS‐CoV‐2 transmission. Ecotoxicol Environ Saf 2020; 202: 111015. 32800237 10.1016/j.ecoenv.2020.111015 PMC7346818

[7] Carraturo F , Del Giudice C , Morelli M *et al*. Persistence of SARS‐Cov‐2 in the environment and COVID‐19 transmission risk from environmental matrices and surface. Environ Pollut 2020; 265: 115010. 32570023 10.1016/j.envpol.2020.115010 PMC7280109

[8] Somerville CC , Shoaib M , Kuschner CE *et al*. Prospective analysis of SARS‐Cov‐2 dissemination to environmental surfaces during endoscopic procedures. Endosc int Open 2021; 9: E701–5. 33937511 10.1055/a-1395-6946 PMC8062224

[9] Gu J , Han B , Wang J . COVID‐19: Gastrointestinal manifestations and potential fecal‐oral transmission. Gastroenterology 2020; 158: 1518–9. 32142785 10.1053/j.gastro.2020.02.054 PMC7130192

[10] Meng XJ , Liang TJ . SARS‐CoV‐2 infection in the gastrointestinal tract: Fecal–oral route of transmission for COVID‐19? Gastroenterology 2021; 160: 1467–9. 33422479 10.1053/j.gastro.2021.01.005 PMC7790455

[11] Kagawa Y , Fukuzawa M , Itoi T . Novel dedicated plastic cube for infection prevention during gastrointestinal endoscopy and endoscopic retrograde cholangiopancreatography. Dig Endosc 2020; 32: 991. 32428286 10.1111/den.13728 PMC7280593

[12] Fukumoto T , Iwasaki S , Fujisawa S *et al*. Efficacy of a novel SARS‐CoV‐2 detection kit without RNA extraction and purification. Int J Infect Dis 2020; 98: 16–7. 32599282 10.1016/j.ijid.2020.06.074 PMC7318955

[13] Han C , Duan C , Zhang S *et al*. Digestive symptoms in COVID‐19 Patients with mild disease severity: Clinical presentation, stool viral RNA testing, and outcomes. Am J Gastroenterol 2020; 115: 916–23. 32301761 10.14309/ajg.0000000000000664 PMC7172493

[14] Chen Y , Klein SL , Garibaldi BT *et al*. Aging in COVID‐19: Vulnerability, immunity and intervention. Ageing Res Rev 2021; 65: 1101205. 10.1016/j.arr.2020.101205 PMC7604159 33137510

[15] Chen N , Zhou M , Dong X *et al*. Epidemiological and clinical characteristics of 99 cases of 2019 novel coronavirus pneumonia in Wuhan, China: A descriptive study. Lancet 2020; 395: 507–13. 32007143 10.1016/S0140-6736(20)30211-7 PMC7135076

[16] Lee S , Yoon GY , Myoung J , Kim SJ , Ahn DG . Robust and persistent SARS‐Cov‐2 infection in the human intestinal brush border expressing cells. Emerg Microbes Infect 2020; 9: 2169–79. 32969768 10.1080/22221751.2020.1827985 PMC7580600

[17] Guan WJ , Ni ZY , Hu Y *et al*. Clinical characteristics of coronavirus disease 2019 in China. N Engl J Med 2020; 382: 1708–20. 32109013 10.1056/NEJMoa2002032 PMC7092819

[18] Jin X , Lian JS , Hu JH *et al*. Epidemiological, clinical and virological characteristics of 74 cases of coronavirus‐infected disease 2019 (COVID‐19) with gastrointestinal symptoms. Gut 2020; 69: 1002–9. 32213556 10.1136/gutjnl-2020-320926 PMC7133387

[19] Gu S , Chen Y , Wu Z *et al*. Alterations of the gut microbiota in patients with coronavirus disease 2019 or H1N1 influenza. Clin Infect Dis 2020; 71: 2669–78. 32497191 10.1093/cid/ciaa709 PMC7314193

[20] Zuo T , Zhang F , Lui GCY *et al*. Alterations in gut microbiota of patients with COVID‐19 during time of hospitalization. Gastroenterology 2020; 159: 944–955.e8. 32442562 10.1053/j.gastro.2020.05.048 PMC7237927

[21] Natarajan A , Han A , Zlitni S *et al*. Standardized preservation, extraction and quantification techniques for detection of fecal SARS‐CoV‐2 RNA. Nat Commun 2021; 12: 5753. 34599164 10.1038/s41467-021-25576-6 PMC8486790

[22] Grivas P , Khaki AR , Wise‐Draper TM *et al*. Association of clinical factors and recent anticancer therapy with COVID‐19 severity among patients with cancer: A report from the COVID‐19 and Cancer Consortium. Ann Oncol 2021; 32: 787–800. 33746047 10.1016/j.annonc.2021.02.024 PMC7972830

[23] Jalkanen P , Kolehmainen P , Häkkinen HK *et al.* COVID‐19 mRNA vaccine induced antibody responses against three SARS‐CoV‐2 variants. Nat Commun 2021; 12: 3991. 34183681 10.1038/s41467-021-24285-4 PMC8239026

[24] Wu Y , Kang L , Guo Z *et al*. Incubation period of COVID‐19 caused by unique SARS‐CoV‐2 strains: A systematic review and meta‐analysis. JAMA Netw Open. 2022; 5: e2228008. 35994285 10.1001/jamanetworkopen.2022.28008 PMC9396366

[25] Chan JFW , Yuan S , Kok KH *et al*. A familial cluster of pneumonia associated with the 2019 novel coronavirus indicating person‐to‐person transmission: A study of a family cluster. Lancet 2020; 395: 514–23. 31986261 10.1016/S0140-6736(20)30154-9 PMC7159286

[26] Amirian ES . Potential fecal transmission of SARS‐Cov‐2: Current evidence and implications for public health. Int J Infect Dis 2020; 95: 363–70. 32335340 10.1016/j.ijid.2020.04.057 PMC7195510

[27] Wu Y , Guo C , Tang L *et al*. Prolonged presence of SARS‐Cov‐2 viral RNA in faecal samples. Lancet Gastroenterol Hepatol 2020; 5: 434–5. 32199469 10.1016/S2468-1253(20)30083-2 PMC7158584

[28] Pamplona J , Solano R , Soler C , Sabat M . Epidemiological approximation of the enteric manifestation and possible fecal‐oral transmission in COVID‐19: A preliminary systematic review. Eur J Gastroenterol Hepatol 2021;33:e21–9. 32956179 10.1097/MEG.0000000000001934

