# Supplementary material for: Effect of COVID‐19 infection on the gastrointestinal tract considering preventive methods during endoscopic procedures
Source: DEN Open. 2023 Aug 27;4(1):e290. doi: 10.1002/deo2.290 (PMC10461040; doi:10.1002/deo2.290)
Supplement: Supplementary file 1 — Figure S1 The severity of cases affected by COVID‐19 was evaluated according to the medical guide of the Ministry of Health, Labour, and Welfare. [file DEO2-4-e290-s001.pdf]

Supplementary figure

Severity of COVID-19	Clinical Oxygen SpO2	Clinical condition
**Mild**	$SpO_2 \geq 96\%$	No respiratory symptoms
		Cough and no respiratory symptoms observed without pneumonia
**Moderate I**<br>**without respiratory disfunction**	$93\% < SpO_2 < 96\%$	Dyspnea, Pneumonia
**Moderate II**<br>**with respiratory disfunction**	$SpO_2 \leq 93\%$	Oxygen administration required
**Severe**		Intensive care unit admission

Ministry of Health, Labour and Welfare Medical Guide
